# Alginate oligosaccharide alleviates senile osteoporosis via the RANKL–RANK pathway in D‐galactose‐induced C57BL/6J mice

**DOI:** 10.1111/cbdd.13904

**Published:** 2021-11-18

**Authors:** Shan Wang, Wenjing Feng, Jianya Liu, Xufu Wang, Lina Zhong, Chengxiu Lv, Meiping Feng, Nina An, Yongjun Mao

**Affiliations:** ^1^ Department of Geriatric Medicine The Affiliated Hospital of Qingdao University Qingdao China; ^2^ Department of Epidemiology and Health Statistics The School of Public Health of Qingdao University Qingdao China; ^3^ Department of Nuclear Medicine The Affiliated Hospital of Qingdao University Qingdao China; ^4^ Department of General Practice Anyang District Hospital of Puyang City Anyang China

**Keywords:** aging, alginate oligosaccharide, D‐galactose, inflammation, osteoporosis

## Abstract

Osteoporosis is a systemic skeletal disorder characterized by reduced bone mineral density (BMD) and bone quality and increased bone porosity, which increase the risk of bone fracture. Inflammation, one of the important mechanisms related to aging, is associated with osteoporosis. Treatment with anti‐inflammatory agents is effective for alleviating senile osteoporosis. Alginate oligosaccharide (AOS) can prevent and treat diseases related to inflammation, oxidative stress, and immunity. This study evaluates the effect of AOS on osteoporosis and investigates the underlying mechanism. Osteoporosis model was induced by D‐galactose (D‐gal) (200 mg kg^−1^ day^−1^) for eight weeks. Three groups were administered via AOS (50, 100, and 150 mg kg^−1^ day^−1^) for four weeks, while a control group received sterile water (5 ml kg^−1^ day^−1^) for 8 weeks. The results showed that AOS improved bone density and bone microstructure in D‐gal‐induced osteoporosis mice. AOS inhibited osteoclast proliferation, probably through the suppression of receptor activator of nuclear factor‐kappa B ligand (RANKL)‐associated nuclear factor kappa B (NF‐κB) and c‐Fos signaling pathway. AOS also increased osteoprotegerin (OPG) expression and competitively inhibited the binding between RANK and RANKL in senile osteoporosis. Further, AOS decreased the secretion of serum osteocalcin and reduced bone conversion. Together, these results demonstrate the anti‐osteoporosis activity of AOS in mice with osteoporosis.

## Graphical abstract

Osteoporosis is a systemic skeletal disorder characterized by reduced bone mineral density (BMD) and bone quality and increased bone porosity, which increase the risk of bone fracture. AOS improved bone density and bone microstructure in D‐gal‐induced osteoporosis mice.

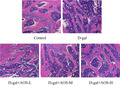

## 1 INTRODUCTION

Osteoporosis, the most common age‐related bone disease (Javaheri & Pitsillides, 2019), is characterized by the destruction of the bone microstructure, resulting in the reduction in the bone strength and an increase in the susceptibility to fracture (Feehan et al., 2019). Homeostasis, the balance between osteoclast‐mediated bone resorption and osteoblast‐mediated bone formation, is lost during aging, leading to osteoporosis (Kiernan et al., 2017; Ponti et al., 2019). Osteoporosis adversely affects the quality of life and has been associated with increased mortality in the elderly population (Qadir et al., 2020). It is regarded as a systemic skeletal disorder related to microarchitectural deterioration, decreased bone mass, and increased fragility and has raised widespread concerns (Chen et al., 2019; Zhao et al., 2019).

Receptor activator of nuclear factor‐kappa B ligand (RANKL) plays an important role in osteoclastogenesis (Zhao et al., 2017) and binds with receptor activator of nuclear factor‐kappa B (RANK), resulting in the recruitment of tumor necrosis factor (TNF) receptor‐associated factors (TRAFs) (Ko et al., 2019). This process subsequently activates the downstream signaling pathways such as NF‐κB, c‐Fos, and mitogen‐activated protein kinase (MAPK) and promotes the activation, differentiation, and maturation of osteoclasts (Zhong et al., 2019). Current research studies highlight the RANKL signaling pathway as the principal target of bone resorptive factor that promotes osteoclast activation and bone loss (Wang et al., 2018). Osteoclasts firmly adhere onto the bone surface and not only produce hydrochloric acid to dissolve bone minerals but also secrete proteolytic enzymes such as cathepsin K to dissolve the bone matrix. Osteoprotegerin (OPG) encoded by the TNFRSF11B gene antagonizes RANKL and inhibits osteoclastogenesis and has been demonstrated to significantly alleviate osteoporosis (Tokunaga et al., 2020). The OPG–RANK–RANKL axis is considered one of the most crucial signaling pathways involved in the regulation of osteoclast differentiation and maturation (Zhu et al., 2019).

Under normal conditions, pathogenic invasion leads to the activation of inflammatory immune responses, which exert protective effects on the body. However, aging is often associated with a low level chronic inflammatory state, also known as “inflammatory aging” (Ponti et al., 2019). Bone aging is a key phenomenon associated with the occurrence and development of osteoporosis, and inflammation may serve as one of the important regulatory mechanisms underlying aging‐related osteoporosis.

Long‐term injection of D‐galactose (D‐gal) in experimental animals leads to a series of pathological changes similar to those observed during natural aging, including cognitive impairment, cardiovascular diseases, and reduced bone mass (Samad et al., 2019). Numerous studies have shown that alginate oligosaccharide (AOS) not only maintains the biological functions of its alginate polysaccharide but also exerts better biological effects. AOS‐mediated inhibition of chronic inflammation could protect mice from D‐gal‐induced myocardial damage through the inhibition of the NF‐κB inflammation pathway (Hu et al.,^2019^). AOS exerts anti‐oxidant (Lane, 2006), anti‐inflammation (Raterman & Lems, 2019), anti‐pathogenic (Falkeborg et al., 2014), and other biological activities. However, most studies have focused on osteoporosis in post‐menopausal women and reports in older men with osteoporosis are scarce. In addition, no studies have investigated the role of AOS in osteoporosis, so we hypothesized that AOS has a protective role in D‐gal‐induced senile osteoporosis and whether it is regulated through the RANKL/RANK/OPG and NF‐KB pathways.

In the present study, we established an osteoporosis model of aging mouse to systemically evaluate the anti‐osteoporotic activity of AOS and investigate the possible underlying mechanism. However, no studies have investigated the role of AOS in osteoporosis, so we hypothesized that AOS has a protective role in D‐gal‐induced senile osteoporosis and whether it is regulated through the RANKL/RANK/OPG and NF‐KB pathways.

## 2 METHODS AND MATERIALS

### 2.1 Alginate oligosaccharide

**FIGURE 1:**
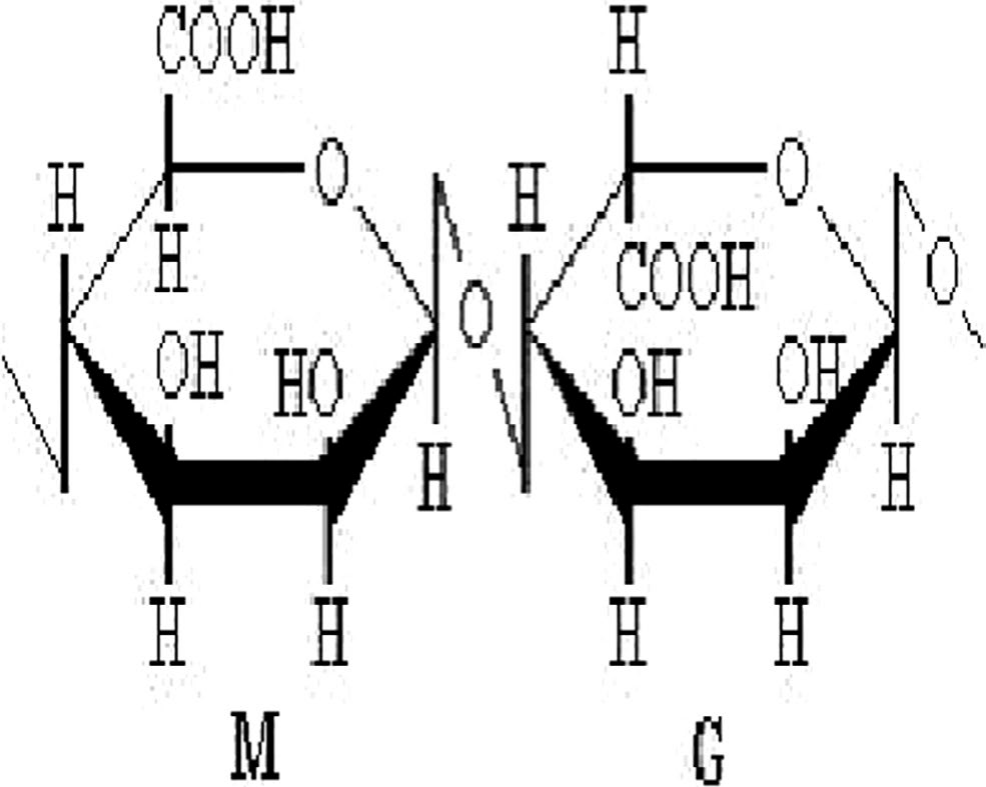
Chemical structure of Alginate oligosaccharide

AOS was purchased from Qingdao BZ Oligo Biotech Co. Ltd. (Qingdao, People's Republic of China). AOS was a new generation of functional oligosaccharides. The oligosaccharides with a polymerization degree of less than 20 were obtained by international leading separation and degradation technology. The chemical structure of AOS obtained by enzymatic degradation is shown in Figure 1.

### 2.2 Animals and groups

We obtained 8‐week‐old C57BL/6J male mice (*n* = 45) from the Jinan Pengyue Experimental Animal Breeding Co., Ltd. (Jinan, China). Mice were housed in an air‐conditioned environment (20℃ ± 2℃) with a 12‐hr light/dark cycle and acclimatized for a week at the Experimental Animal Center of Qingdao University Medical Department. All animal experimental procedures were performed as per the “Guide for the Care and Use of Laboratory Animals” published by the US National Institutes of Health and Public Health Service policy on Humane Care and Use of Laboratory Animals. These mice were randomly divided into five groups as follows: control group (*n* = 9); D‐gal group (*n* = 9); D‐gal + AOS‐L (50 mg kg^−1^ day^−1^, *n* = 9); D‐gal + AOS‐M (100 mg kg^−1^ day^−1^, *n* = 9); and D‐gal +AOS‐H (150 mg kg^−1^ day^−1^, *n* = 9). Mice from the control group were subcutaneously administrated with sterile water (5 ml kg^−1^ day^−1^) for 8 weeks. The mice from the other groups were subcutaneously injected with D‐gal (200 mg kg^−1^ day^−1^) for 8 weeks. From fifth week onward, the mice from D‐gal + AOS‐L, D‐gal + AOS‐M, and D‐gal + AOS‐H groups were treated with 50, 100, and 150 mg kg^−1^ day^−1^ alginate oligosaccharide by oral gavage. The D‐gal and control groups were treated with distilled water (10 ml kg^−1^ day^−1^) by oral gavage for 4 weeks.

### 2.3 Bone mineral density (BMD) analysis

We carefully remove the soft tissue surrounding the proximal femur. The BMD of femur was measured by dual‐energy X‐ray absorptiometry (DEXA; Osteosys Primus, Korea) at a scan pitch of 1.5 mm and a speed of 60 mm/s. The entire femur was evaluated by DEXA for BMD measurement.

### 2.4 Hematoxylin and eosin (H&E) staining

Any histomorphological variation in bone tissue was evaluated using H&E staining. The ipsilateral femur samples were fixed in 4% paraformaldehyde for 4 days and decalcified with a 10% tetrasodium‐ethylenediaminetetraacetic acid (EDTA) aqueous solution for 30 days. The tissues were embedded in paraffin, and the paraffin blocks were sliced to obtain 4‐mm‐thick sections for histological examination. Regions were deparaffinized, hydrated, and stained with H&E. The slices were evaluated under a light microscope (DP73; Olympus) at 200× magnification. The trabecular bone was also assessed.

### 2.5 Western blot analysis

The femur tissue was placed in a burnisher and treated with liquid nitrogen. Femur proteins were extracted using radioimmunoprecipitation assay (RIPA) lysis buffer (Beyotime Biotechnology, Shanghai, China), phenylmethylsulfonyl fluoride (Sigma‐Aldrich, St. Louis, USA), protease inhibitor cocktail (1:100, Sigma‐Aldrich, St. Louis, USA), and phosphatase inhibitor cocktail (1:100, MedChemExpress, NJ, USA). The remaining protein samples were mixed with a sodium dodecyl sulfate polyacrylamide gel electrophoresis (SDS‐PAGE) loading buffer (5×) at a ratio of 4:1 and boiled for 5 min. The proteins were separated by PAGE at 80 V voltage for about 30 min, followed by 110 V for 1 hr. The separated protein bands were transferred onto polyvinylidene fluoride (PVDF) membranes (Bio‐Rad, USA), which were then washed with Tris‐buffered saline with Tween‐20 (TBST) for 10 min and placed in a blocking buffer (TBST and 5% nonfat dry milk) at room temperature for 2 hr. The membranes were washed with TBST for 10 min and incubated with antibodies such as p53 (1:1,000; Abcam, USA), NF‐κB (1:1,000; Santa Cruz Biotechnology, USA), IκB‐α (1:1,000; Santa Cruz Biotechnology, USA), β‐actin (1:2000, Abcam, UK), Lamin‐B (1:2000; Santa Cruz Biotechnology, USA), C‐Fos (1:1,000; Elabscience, China), and RANKL (1:;1,000; Elabscience, China) antibodies overnight at 4℃. Following incubation, the membranes were washed with TBST for 45 min and probed with horseradish peroxidase (HRP)‐conjugated secondary antibodies in primary buffer (1:5,000; Elabscience Biotechnology, China). The membranes were treated with enhanced chemiluminescence reagents (Millipore, USA) for 1 min and exposed to chemical luminescent imager (Vilber Lourmat, France). The gray value on each strip was calculated using Quantity One software (Bio‐Red, USA).

### 2.6 Extraction of nuclear and cytoplasmic proteins

Nuclear and cytoplasmic protein extraction was carried out after homogenizing the tissues with a tissue homogenizer. Protein extraction was performed as per the protein extraction kit (Solarbio, China) instructions.

### 2.7 Reverse transcription‐quantitative polymerase chain reaction (RT‐qPCR)

The total RNA was isolated from the diaphysis using TRIzol reagent (Life Technologies, Carlsbad, CA, USA) and reverse transcribed into cDNA using a cDNA synthesis kit (Roche, Mannheim, Germany) according the manufacturer's protocol. The primer sequences for genes are shown in Table 1. Real‐time PCR analysis was performed using LightCycler^®^ 96 System (Roche, Indianapolis, IN, USA). The reaction conditions were as follows: 95℃ for 10 min, 95℃ for 10 s, and 60℃ for 10 s and 72℃ for 15 s for 40 cycles. The relative changes in the transcript levels were analyzed according to the 2^−ΔΔCt^ method using β‐actin as the control.

**TABLE 1:** Primer sequences for RT‐qPCR

Gene name		Primer sequence
*RANK*	Forward	CAACTCAACGGATGGCTACACAGG
	Reverse	GCTGGCTGCTTCACTGG
*OPG*	Forward	CACAGAGCAGCTCCGCATCTTG
	Reverse	AAGTGCTTGAGTGCGTACATCAGG

### 2.8 Enzyme‐linked immunosorbent assay (ELISA)

After induction of anesthesia, blood samples were obtained before the mice were sacrificed. The concentrations of osteocalcin in the serum were measured using ELISA kits (Elabscience Biotechnology Co. Ltd., Wuhan, China). These experiments were carried out strictly as per the manufacturer.

### 2.9 Statistical analysis

Statistical analysis was performed using the SPSS statistical software (SPSS, Chicago, USA). Differences between samples were analyzed using one‐way analysis of variance (ANOVA). Two groups with obvious differences were evaluated by the Student's *t*‐test. A value of *p* < .05 was considered significant.

## 3 RESULTS

### 3.1 AOS treatment alleviated the pathological changes of osteoporosis in D‐gal‐induced osteoporosis mice

**FIGURE 2:**
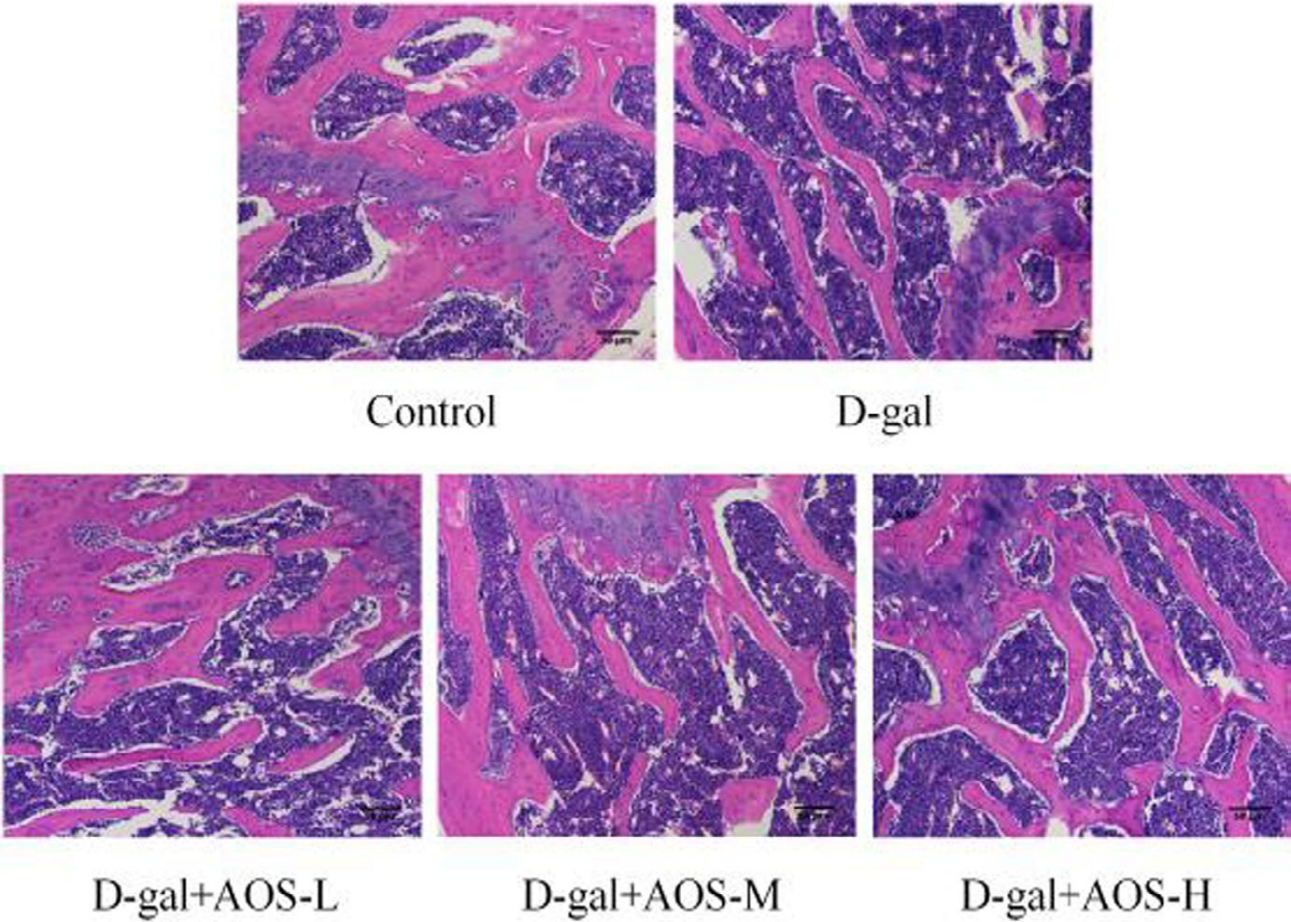
Effects of AOS on the morphology of femur osseous tissues in D‐gal‐induced osteoporosis mice. H&E staining was used to investigate the morphological changes in the femur tissue (Original magnification, ×200)

In the control group, the bone trabecular was dense and compact (Figure 2). As seen in Figure 2, the mice from D‐gal group showed a large bone marrow cavity and had sparse and slender bone trabecular as compared with the mice from the control group. However, the administration of AOS at a dose of 50, 100, and 150 mg kg^−1^ day^−1^ for 4 weeks resulted in the amelioration of the pathological changes of osteoporosis. The trabecular bone was more compact and thicker than that observed for the mice from D‐gal group.

### 3.2 AOS treatment restored the BMD in D‐gal‐induced osteoporosis mice

**FIGURE 3:**
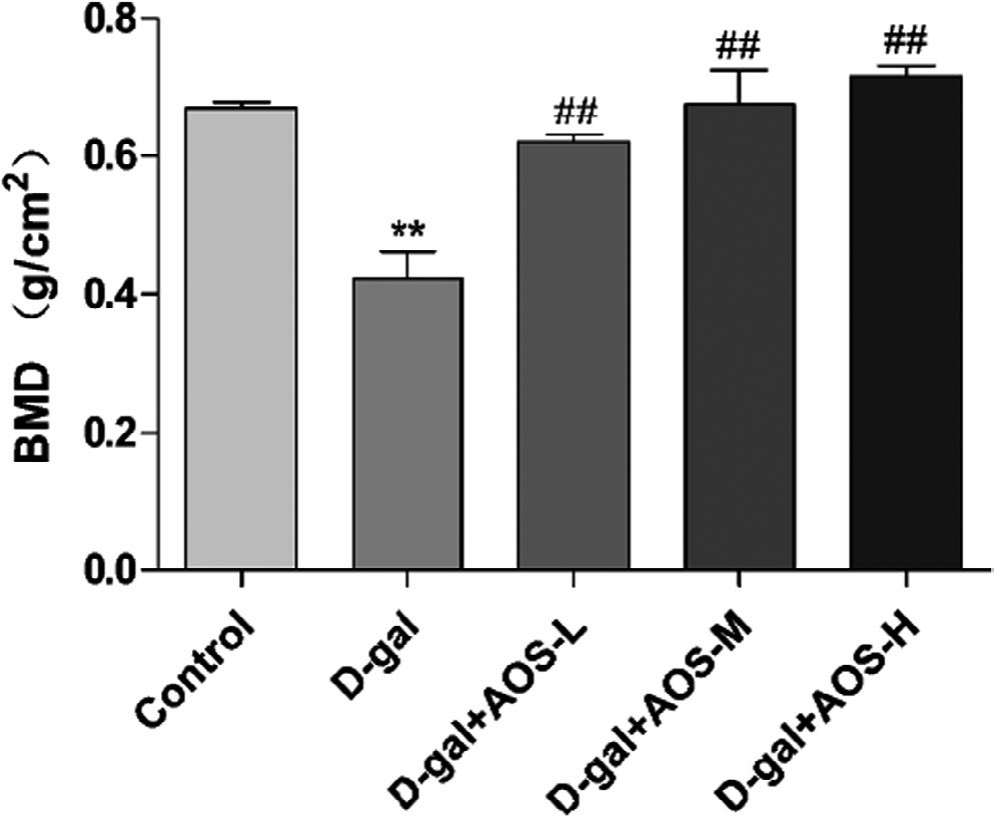
Effect of AOS on the BMD of femur osseous tissues in D‐gal‐induced osteoporosis mice. *p < .05 and **p < .01 versus control mice; #*p* < .05 and ##*p* < .01 versus D‐gal mice

As shown in Figure 3, the 8‐week treatment with D‐gal resulted in a decrease in the femur BMD as compared with the control treatment. However, AOS injection significantly increased the femur BMD in mice with D‐gal‐induced osteoporosis as compared with AOS‐untreated mice.

### 3.3 AOS decreased the serum level of osteocalcin in D‐gal‐induced osteoporosis mice

**FIGURE 4:**
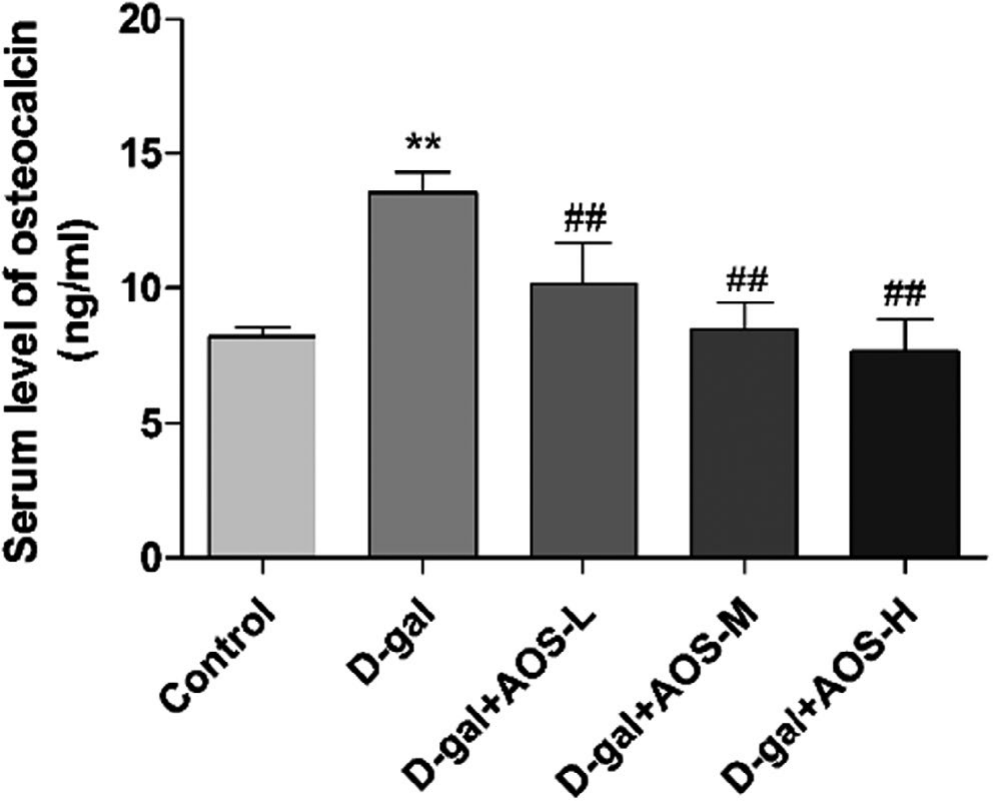
Effect of AOS on the serum level of the osteocalcin in D‐gal‐induced osteoporosis mice. ELISA analysis of osteocalcin level in the serum. *p < .05 and **p < .01 versus control mice; #*p* < .05 and ##*p* < .01 versus D‐gal mice

Figure 4 shows that the level of serum osteocalcin in aging‐related osteoporosis mice increased after D‐gal injection as compared with that observed in control mice. AOS treatment decreased the level of osteocalcin in the serum in a dose‐dependent manner as compared with D‐gal treatment.

### 3.4 AOS downregulated the level of the senescence biomarker p53 in D‐gal‐induced osteoporosis mice

**FIGURE 5:**
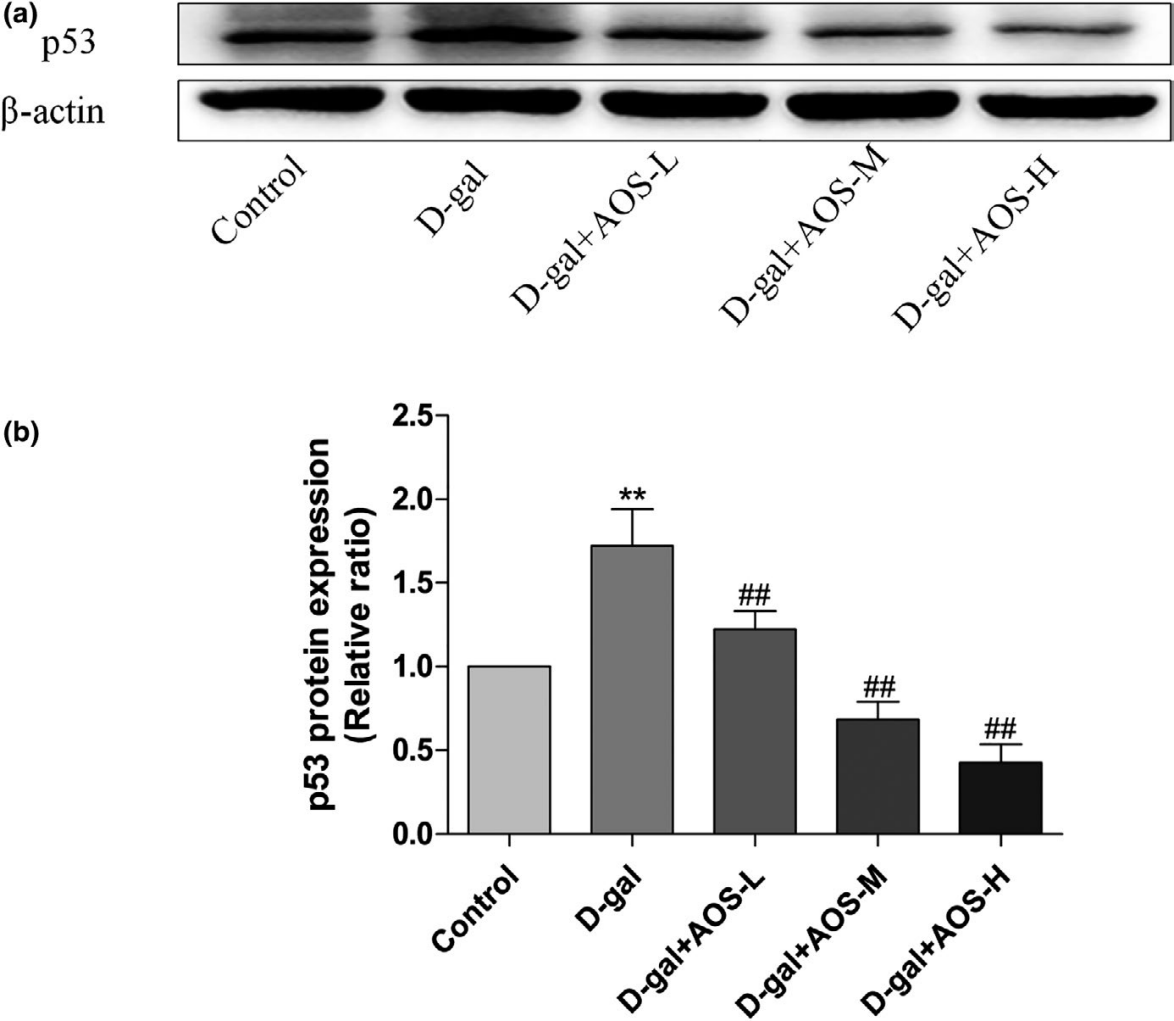
Effect of AOS on the expression level of p53 in the femur tissue of D‐gal‐induced osteoporosis mice. *p < .05 and **p < .01 versus control mice; #*p* < .05 and ##*p* < .01 versus D‐gal mice

Aging results in the gradual increase in the expression level of p53. As shown in Figure 5, the subcutaneous administration of D‐gal significantly increased the level of p53 as compared with the control treatment, while AOS exposure downregulated p53 expression in a dose‐dependent manner.

### 3.5 AOS inhibited the activation of the RANKL/RANK/C‐Fos pathway in femur of D‐gal‐induced osteoporosis mice

**FIGURE 6:**
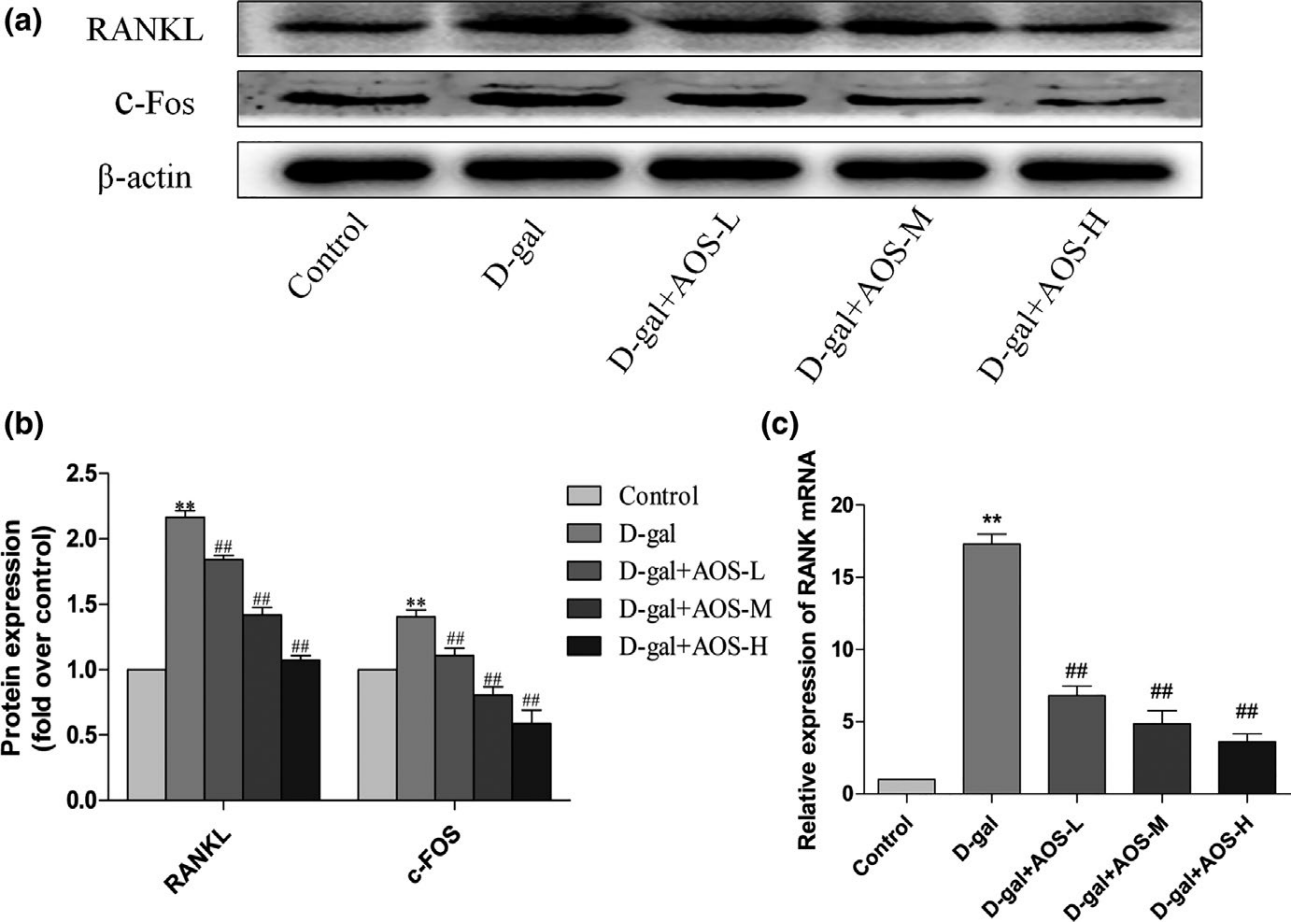
Effect of AOS on the RANKL/RANK/C‐Fos pathway in the femur tissue of D‐gal‐induced osteoporosis mice. (a, b) The expression of RANKL and c‐Fos in the femur tissue was analyzed by Western blotting. (c) The level of RANK mRNA in the femur tissue as assessed with RT‐qPCR is shown. *p < .05 and **p < .01 versus control mice; #*p* < .05 and ##*p* < .01 versus D‐gal mice

The RANKL/RANK/C‐Fos pathway is associated with bone resorption and osteoclast differentiation. We investigated whether AOS participates in the RANKL/RANK/C‐Fos pathway of osteoporosis. As shown in Figure 6, the subcutaneous administration of D‐gal significantly increased the expression levels of RANKL and C‐Fos as compared with the control treatment in the Western blot analysis. However, RANKL and C‐Fos expression levels obviously decreased in the in the femur tissues from D‐gal + AOS‐L, D‐gal + AOS‐M, and D‐gal + AOS‐H mice. The expression of RANK in the femur was evaluated with RT‐qPCR. D‐gal injection induced aging‐related osteoporosis and significantly increased the level of RANK mRNA as compared with the control treatment. However, AOS dose‐dependently downregulated the mRNA expression of RANK.

### 3.6 AOS inhibited the activation of the RANKL/RANK/NF‐κB pathway in femur of D‐gal‐induced osteoporosis mice

**FIGURE 7:**
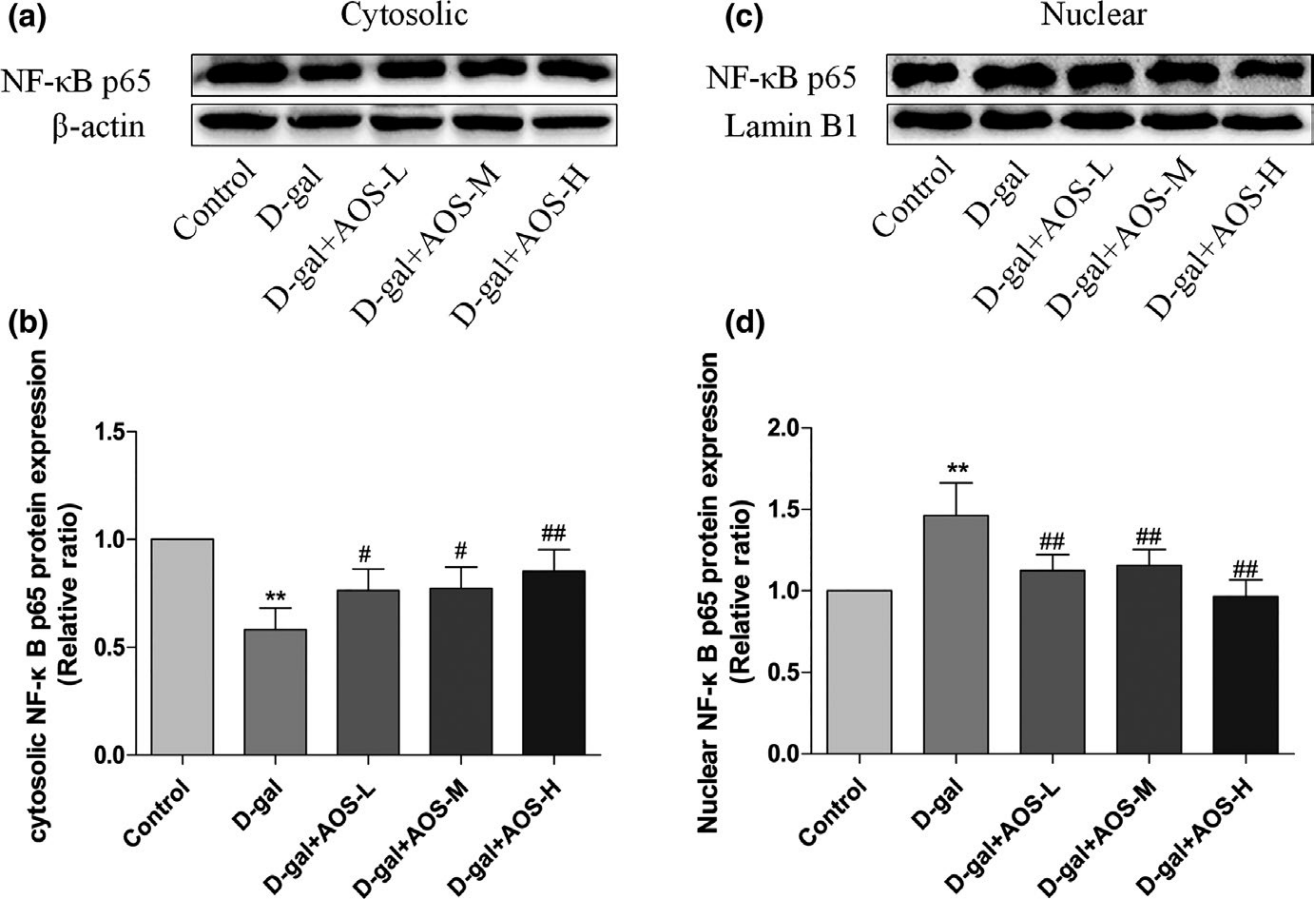
Effect of AOS on the nuclear translocation of NF‐κB in the femur tissue of D‐gal‐induced osteoporosis mice. (a, b) The expression of cytoplasmic NF‐κB p65 in the femur tissue was analyzed by Western blotting. (c, d) The expression of nuclear NF‐κB p65 in the femur was determined by Western blotting. *p < .05 and **p < .01 versus control mice; #*p* < .05 and ##*p* < .01 versus D‐gal mice

**FIGURE 8:**
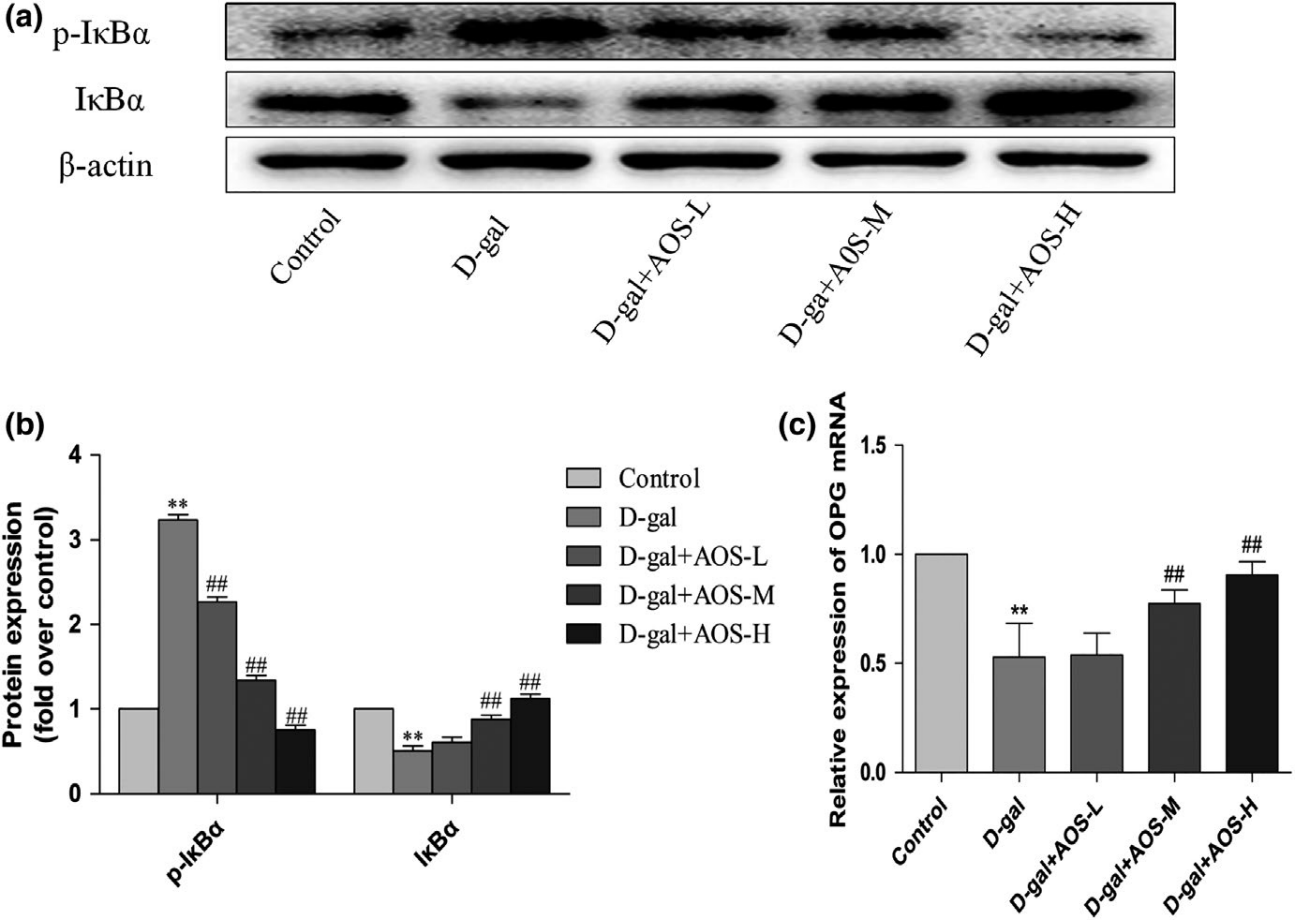
Effect of AOS on the phosphorylation of IκB‐α and OPG expression in the femur tissue of D‐gal‐induced osteoporosis mice. (a, b) The phosphorylation of IκB‐α in femur was determined by Western blot analysis. (c) Effect of AOS on OPG expression in the femur tissue of D‐gal‐induced osteoporosis mice. *p < .05 and **p < .01 versus control mice; #*p* < .05 and ##*p* < .01 versus D‐gal mice

NF‐κB is an important downstream factor of RANK. We examined the nuclear translocation of NF‐κB and the phosphorylation of IκB‐α in the femur tissue by Western blotting. As shown in Figure 7, D‐gal group showed a significant decrease in the expression level of cytoplasmic NF‐κB p65 and an increase level of nuclear NF‐κB p65 in the femur tissue as compared with those from the control group. The administration of AOS markedly increased the level of cytoplasmic NF‐κB p65 and decreased the level of nuclear NF‐κB p65 as compared with D‐gal treatment. This result indicated that AOS significantly inhibited D‐gal‐induced nuclear translocation of NF‐κB in the femur tissue. As shown in Figure 8a, b, we also detected the phosphorylation of IκB‐α in the femur tissue and found that D‐gal significantly promoted IκB‐α phosphorylation as compared with the control treatment. However, AOS administration dose‐dependently inhibited the phosphorylation of IκB‐α in the femur as compared with D‐gal treatment. These results demonstrate the suppressive effect of AOS on the activation of the RANKL/RANK/NF‐κB pathway in femur of D‐gal‐induced osteoporosis mice.

### 3.7 AOS upregulated the expression of OPG in the femur tissue of D‐gal‐induced osteoporosis mice

OPG may bind to RANKL and decrease its expression, thereby alleviating osteoporosis. As shown in Figure 8c, the mice exposed to D‐gal showed a significant decrease in the expression level of OPG as compared with the control mice. However, the level of OPG significantly increased in the femur of the mice from D‐gal + AOS‐L, D‐gal + AOS‐M, and D‐gal + AOS‐H groups. Thus, AOS could dose‐dependently increase the expression of OPG in the femur tissue of D‐gal‐induced osteoporosis mice.

## 4 DISCUSSION

Osteoporosis is the primary cause of mortality and morbidity among aging men (Javaheri & Pitsillides, 2019). Thus, early prevention, diagnosis, and treatment of osteoporosis are extremely important, highlighting the need to improve our understanding about the molecular mechanism underlying osteoporosis for the development of efficient drugs. In addition, it is imperative to develop drugs with minimum side‐effects to reverse the trend in the bone loss. Current treatment regimens for osteoporosis involve anti‐resorptive therapies (Yang, Jiang, et al., 2019), such as bisphosphonates, calcitonin, and estrogen, that inhibit bone resorption and maintain bone mass as well as anabolic therapies such as sclerostin antibody, Dickkopf‐1 antibody, and calcilytic agents that enhance bone formation (Gong et al., 2016). However, these pharmacological agents exhibit some side‐effects such as hypercalcemia, increased risk of breast and endometrial cancers, and gastrointestinal intolerance against bisphosphonate (Javaheri & Pitsillides, 2019). Although there have been many advancements in the diagnosis and management of osteoporosis, contemporary treatment strategies can no longer be considered as ideal owing to their side‐effects and long‐term safety issues. Hence, safer alternatives from natural foods are being explored for the treatment of osteoporosis.

Osteoporosis is a severe health problem among the aging population (Yang, Chen, et al., 2019), as it weakens the bone structure and causes fractures. Alginate, extracted from the marine brown algae, is an acidic polysaccharide that has been extensively studied for its non‐immunogenic and non‐toxic characteristics (Lombardi et al., 2019). Previous studies have shown that alginate produces AOS after depolymerization; AOS exhibits various pharmacological activities such as anti‐proliferative (Tajima et al., 1999), anti‐inflammatory (Zhou et al., 2015), anti‐apoptotic (Guo et al., 2016), and anti‐oxidative effects (Guo et al., 2017). In the present study, we investigated the effects of AOS on D‐gal‐induced osteoporosis in mice. Under normal conditions, mouse femur was dense and orderly arranged. However, D‐gal treatment resulted in the enlargement of the bone marrow cavity and sparse and thin trabeculae; large fragments were broken and poorly arranged. AOS administration increased the thickness of the periosteum and resulted in a thick, dense trabecular bone. In particular, the pathological changes in the mice treated with AOS at a dose of 150 mg/kg were almost similar to those reported in control mice. These results demonstrate the beneficial effects of AOS in the prevention of bone loss and protection of bone microstructure induced by aging.

The D‐gal‐induced aging mouse model was used herein to investigate the effect of AOS on senile osteoporosis and evaluate the possible underlying mechanism to provide a new theoretical basis and intervention target to delay osteoporosis. Studies have shown that the continuous injection of D‐gal in animals may accelerate the aging process, consistent with the symptoms of natural aging after a period of time (bone disease, cardiac dysfunction (Tusi et al., 2011), memory impairment (Wang et al., 2007), decrease in life expectancy, and muscle shrinkage (Jeremy et al., 2019). The mechanism underlying D‐gal‐induced aging may involve various phenomena such as elevated pro‐inflammatory factors, accumulation of reactive oxygen species (ROS), mitochondrial dysfunction, and DNA damage.

RANKL, a member of the TNF receptor family, is generated by osteoblasts/osteocytes and binds to the RANK receptor, resulting in the activation of osteoclast differentiation (Wang et al., 2019). NF‐κB is involved in the RANKL‐mediated osteoclastogenesis and plays a significant role in osteoclast differentiation (El‐Baz et al., 2019; Krzysztoforska et al., 2019). Recent studies have shown that NF‐κB is involved in osteoporosis and that the inhibition of NF‐κB results in the alleviation of osteoporosis in aging mice (Tilstra et al., 2011). Our data reveal that AOS regulates RANKL‐mediated osteoclastogenesis possibly through the inhibition of NF‐κB activation via inhibiting the phosphorylation of IκB‐α and nuclear translocation of NF‐κB p65. Moreover, AOS could suppress osteoclastogenesis by downregulating C‐Fos, which is the downstream molecule of RANKL. We also show that AOS could possibly alleviate osteoporosis by upregulating the expression of OPG, which is known to inhibit osteoporosis.

Osteocalcin is a non‐collagenous vitamin K‐dependent protein. Osteocalcin may recruit osteoclast precursors and promote the differentiation of osteoclast precursors into osteoclasts, suggesting that osteocalcin may be a new target for the treatment of osteopenia or osteoporosis (Lambert et al., 2016). This study demonstrates that the administration of AOS reduced the activity of osteoclasts, inhibited bone resorption, and improved osteoporosis by reducing osteocalcin level. This is the first study to demonstrate the effects of AOS on senile osteoporosis and bone metabolism. In conclusion, the inflammation promotes the progress of osteoporosis by the RANKL/RANK‐related pathway and anti‐inflammation can alleviate the development of osteoporosis. Meanwhile, the administration of AOS reduced the activity of osteoclasts, inhibited bone resorption, and improved osteoporosis and its mechanisms may be related to RANKL/RANK signaling pathway. Nevertheless, our conclusion based on the mice model of osteoporosis. Furthermore, the specific mechanism of AOS in osteoporosis should be furthermore investigated.

## CONFLICT OF INTEREST

The authors declare that they have no competing interests.

## Data Availability

The data sets generated and analyzed during the study are available from the corresponding author on request.
